# Supplementary material for: Constraints on axion-like dark matter from a SERF comagnetometer
Source: Nat Commun. 2023 Sep 18;14:5784. doi: 10.1038/s41467-023-41162-4 (PMC10507093; doi:10.1038/s41467-023-41162-4)
Supplement: Supplementary file 1 — Supplementary Information [file 41467_2023_41162_MOESM1_ESM.pdf]

Supplementary Material

CONTENTS

S1. Calibration of the EPR Resonance 2

S2. Noise Estimation of the Detector 2

S3. Likelihood function 3

S4. Statistical Excess and Second stage analysis 4

S1. CALIBRATION OF THE EPR RESONANCE

In this section, we detail the way in which we track and account for time-variation in the response function of the sensor, primarily due to decay of the helium polarization during a particular run.For each run at a given constant axial magnetic field, we characterize the response function to oscillatory magnetic field measured before and after the recording, denoted with subscript pre and post respectively. These two measured response functions, are denoted by $Y_{\rm pre}(f)$ ($Y_{\rm post}(f)$) and are characterized in the frequency domain in units of V/T. For clarity, the black curves exemplified in Fig. 3 are the $Y_{\rm pre}(f)$ functions at two different magnetic fields. We construct them by measuring the response to oscillatory transverse magnetic fields at several $(10-30)$ sampled points and interpolate between the sampled points. We characterize their resonance frequency by the location of their peak response $f_{\rm pre}$ and $f_{\rm post}$ as well as the linewidth $\Gamma_{\rm pre}$ and $\Gamma_{\rm post}$. Using these parameters, we can describe the corresponding normalized response functions $y_{\rm pre}$ and $y_{\rm post}$ using a single unitless parameter η

$$y_{\rm pre}(\eta) = Y_{\rm pre}(\eta \cdot \Gamma_{\rm pre} + f_{\rm pre}) / \max(Y_{\rm pre}), \tag{1}$$

$$y_{\rm post}(\eta) = Y_{\rm post}(\eta \cdot \Gamma_{\rm post} + f_{\rm post}) / \max(Y_{\rm post}). \tag{2}$$

The normalized functions are a shifted and rescaled version of their parent response functions. They are defined such that each normalized response has a maximal unity response at $\eta = 0$ and a width of 1.

We construct the estimated amalgam during the experiment $Y_x(f)$ as a linear interpolation between the initial and final measured responses. We use a single parameter $x(t) \in [0, 1]$ and take the following interpolation

$$Y_x(f) = Y_{\max}(x) \left(x y_{\rm pre} \left(\frac{f - f_{\rm res}(x)}{\Gamma(x)} \right) + (1-x) y_{\rm post} \left(\frac{f - f_{\rm res}(x)}{\Gamma(x)} \right) \right), \tag{3}$$

using the definitions

$$Y_{\max}(x) = x \max(Y_{\rm pre}) + (1-x) \max(Y_{\rm post}) \tag{4}$$

$$\Gamma(x) = x \Gamma_{\rm pre} + (1-x) \Gamma_{\rm post} \tag{5}$$

$$f_{\rm res}(x) = x f_{\rm pre} + (1-x) f_{\rm post}. \tag{6}$$

It is constructed to be a weighted average between two functions of the same central value, width and height. The weight, height of the central value of the function is the weighted sum of the weights, heights and central values of the two measurements respectively, where the only free parameter is the how to weight the two measurements. To observe the slow change of x in a continuous manner, we estimate the value of x every $1-5$ minutes by taking the absolute value of the Fourier transform of the measured data in a window of about 1 second and fitting to the amalgam Y_x. Importantly, this low-resolution procedure does not resolve the ALP spectrum and does not introduce bias to the measurement. We also note that $Y = 0$ was taken outside the measured frequency window, thus only underestimating the sensitivity of the detector in this exclusion search.

While the interpolation of the EPR calibration between the pre- and post-calibration measurements is somewhat arbitrary, we find that our measurements are relatively insensitive to this choice. We have tested various interpolation methods and found that any reasonable modification produces very similar results. Fig. S1 illustrates what the interpolation looks like for a dozen different values of B_z. All 110 pairs of calibration measurements used in our search are available at Ref. [57], together with the additional calibration measurements of those not used in limit setting. A tabulated list of all external magnetic fields used can be found in Table. S1.

As can be seen in Fig. S1, the response at higher frequencies ($\gtrsim 5$ kHz) follows a Lorentzian profile as one would expect from a direct measurement of the left hand side of Eq. (4). However, at low frequencies, frequency dependence of the electrical filter used for the measurement, as well as interference from the lorentzian centered around negative frequencies, lead to deviations from the Lorentzian profile. Our calibrations account for these deviations, with uncertainty of about $\lesssim 10\%$ at lower frequencies ($\lesssim$ kHz), estimated by comparing the response at a given frequency to the one predicted by a linear fit to its neighboring measured responses. Pre-calibration measurements at low fields ($\lesssim 1.2 \mu T$), have an additional deviation from the lorentzian profile, due to a small but non-negligible decay of the helium during the measurement. By comparing pre- and post- calibrations with the same resonance frequency, we may see that this causes up to $\sim 20\%$ change in the fitted Γ. Since the measurements at low magnetic fields are much longer than the T_1 of the helium, the response is dominated by the response after the helium reaches its steady state. And, since $|f_{\rm pre} - f_{\rm post}| \sim \Gamma$ this has an altogether a minor effect.

S2. NOISE ESTIMATION OF THE DETECTOR

We study the noise profile of the detector in our experiment by be examining its spectral shape. Except for a few spikes that can result from oscillating stray fields, to leading order the noise is a combination of spin-dependent noise, and a technical noise. The

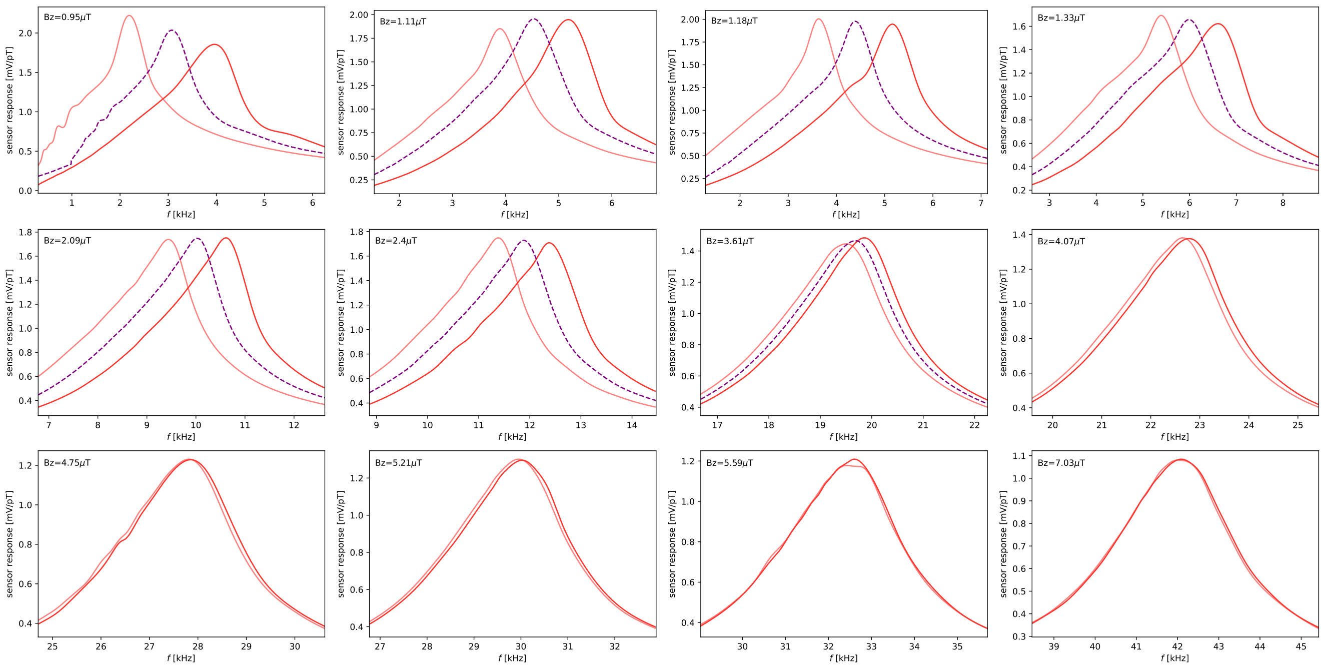

FIG. S1. **Sensor response to oscillating transverse magnetic fields at different external axial magnetic fields**. Each plot has the value of the applied B_z in an inline label. The bright red lines correspond to the pre-recording calibration measurements, while the darker reds correspond to the post-recording calibrations. For measurements in which the response has changed significantly during data-taking, we have interpolated the change of calibration as a function of time. As an example, the dashed purple line shows the middle interpolated curve for the time at which the peak of the response was the mean of the post- and pre-calibrated peak responses. The full set of calibration plots can be found in Ref. [57].

former, takes the spectral shape of our response function (which for some frequencies is nearly Lorentzian), as shown in Fig. S1 for different values of B_z. This is spectral noise structure that is expected e.g., from magnetic field noise. The technical noise on the other hand, appears as an offset whose spectral dependence is much smaller, and can result from Photon shot noise of the probe beam and electronic noise at the detector which are mostly independent of the spin polarization. Using our estimation to the technical noise in Fig. 4 in the main text, we use the calibration measurements and provide an estimate to the amplitude of the magnetic noise.

For that purpose, we take fifteen different measurements at different values of B_z, each fives seconds long, and fit the power spectral density (PSD) to the fitting function $B_{\text{peak}}^2 Y_x^2(f) + N^2(f)$ where $N(f)$ is the technical noise floor, $Y_x(f)$ is the calibrated response function and B_{peak} is the free fitting parameter designating the amplitude of noise associated with magnetic fields. In Fig. S2, we present the (square root of) the raw PSD in black and the fitted noise spectrum in blue. To avoid being strongly dependent on narrow peaks, we use a 2-Hz wide running median filter on the full noise spectrum, and a 0.2-Hz wide running median filter on the technical noise offset. This procedure yields magnetic field noise that varies between $4.9 - 7.6$ fT/$\sqrt{\text{Hz}}$.

S3. LIKELIHOOD FUNCTION

Our analysis constructs and uses the same likelihood functions presented in detail in the Supplementary Material in Ref. [14], except for a different construction of the sensitivity matrices α, which we detail in this section.

The matrices α link the ALP vector $\mathcal{A}/\sqrt{2\rho_a}$ at a given frequency with the data vector $\mathbf{d}$ in the frequency domain where $\rho_a = 0.4\ \text{GeV}/\text{cm}^3$. In this work, the rotating-wave approximation is not assumed, and the contribution of negative frequencies is explicitly considered. Denoting the full sensitivity matrices by α^Σ, their matrix element is given by

$$\alpha^\Sigma_{mn,i} = (\alpha_i(f_m, f_n) + \alpha_i^*(-f_m, -f_n)), \tag{7}$$

where each $\alpha_i(f_m, f_n)$ is computed by integrating over the response

$$\alpha_i(f_m, f_n) = \frac{\sqrt{2\rho_\text{a}}}{T} \int_0^T dt\; g_\text{eff}(f_n, t) e^{2i\pi(f_n - f_m)t} \Xi(t)(\hat{x}(t) + i\hat{y}(t))_i \mathcal{Y}_{x(t)}(f_n). \tag{8}$$

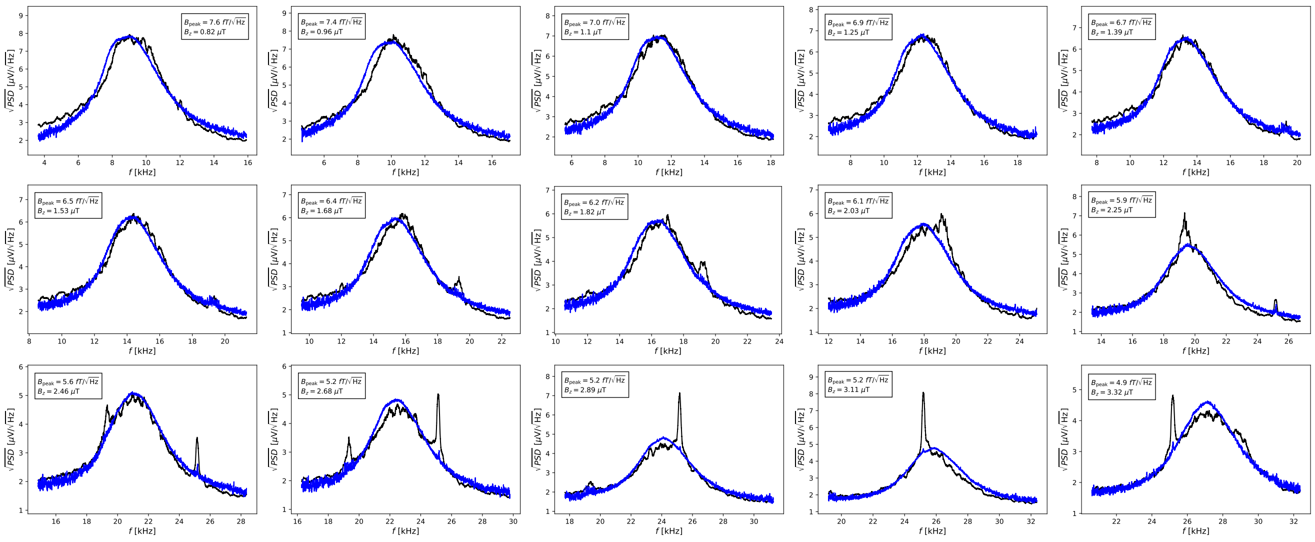

FIG. S2. **Magnetic noise spectral density of individual measurements.** Each subplot corresponds to a different axial magnetic field B_z and a different EPR resonance. The black curve corresponds to the measured square root of the Power Spectral Density (PSD), while the blue curve is a fit to an incoherent sum of the magnetic response function plus the technical noise offset, estimated independently from other measurements. The fitted data has been smoothed using running median filters (see text for further details), to reduce the effect of narrow peaks on the fit, which is meant to capture the wider features. The good overlap between the noise structure and the magnetic calibration response validates that the noise originates from the spins and is likely dominated by magnetic noise. The amplitude of the fit, denoted as B_{peak} in each subplot, provides the magnetic noise floor, which varies between 4.9–7.6 fT/$\sqrt{\text{Hz}}$ in these measurements. The label shows B_{peak}, the value of the magnetic noise contribution to the measured noise at the peak in units of magnetic field noise.

Here t is the time from the beginning of the measurement, and $\hat{x}(\hat{y})$ is the direction of the probe beam (the direction perpendicular to the probe and pump beams), projected to the ith direction in galactic coordinates (relying on Ref. [44]). T is the total measurement time, and f_m, f_n are two frequencies in the fourier series of the data in the $[0, T]$ interval. $g_{\text{eff}}(f_n, t)$ is defined in Eq. 8 of the main text, and $\Xi(t)$ is the binary quality cut function ($\zeta(t)$ in Ref. [14]). We define $\mathcal{Y}_{x(t)}(f)$ below in Eq. 9, and it represents the response to magnetic fields to a single (positive or negative) frequency f.

The measured response function $Y_x(|f|)$ originates from an input sinusoidal function, which includes both positive and negative frequencies. To construct the response function that explicitly differentiates between positive and negative frequencies, we assume that the response is a sum of two Lorentzian functions centered around $\pm f_{\text{res}}(x)$ with width $\Gamma(x)$. We further allow the two Lorentizan functions to have any positive common scaling factor that depends on $|f|$, to allow for any frequency dependent response of the electronics of the measurement circuits. We then find that the modified response is

$$\mathcal{Y}_x(f) = Y_x(|f|) \left| \frac{\Gamma + if + if_{\text{res}}}{\Gamma + if_{\text{res}}} \right| \frac{\Gamma + i(f_{\text{res}} - f)}{|\Gamma + i(f_{\text{res}} - f)|}. \tag{9}$$

The second term produces the correct phase for the response, and is only important when f_{res} crosses f. We use a slightly simpler function for the phase in our numerical calculations that reproduces its time dependence.

S4. STATISTICAL EXCESS AND SECOND STAGE ANALYSIS

While the pre-unblinding procedure aimed to exclude possible new interactions, we found that several thousand spectral points are inconsistent with our white noise model and show statistical excess. We first define the statistical excess threshold from the calculated test-statistic. Because ALP signals at different frequencies are nearly independent, the number of independent tests is large, and the look-elsewhere effect needs to be taken into account. We adopt the estimation by Ref. [34] to determine the threshold and find it to be 51 for our mass-range. We note that in computing their threshold, Ref. [34] uses a numerical factor which they find using a monte-carlo tailored to their specific set of measurements. This parameter should be different for this work, but the threshold is sub-logarithmically dependent on this $\mathcal{O}(1)$ parameter, and therefore even major changes would only change the threshold very minutely. This value is equivalent to a 5-sigma global excess (the threshold for the test statistic is twice the log-likelihood ratio of the best-fit point and the background-only hypothesis).

With this definition of an excess, we logarithmically binned the data with bins of size 10^{-6} in mass. The number of bins with an excess is an estimate for the number of independent ALP candidates. Using this method, we found that out of approximately 5 million bins, 3701 (3676) independent candidates in the ALP-proton (ALP-neutron) analysis showed a statistical excess. Most of these candidates overlapped between the two analyses, resulting in a total of 3788 different independent candidates when combining the two datasets. The few non-overlapping bins are those where one of the two analyses was slightly below the threshold and the other slightly above.

To gain a better understanding of their nature, we conducted a second analysis post-unblinding, which revealed that the majority of the peaks are inconsistent with an ALP-induced signal. First, we reanalyzed the data using an additional dataset that was not used during the limit-setting phase. As stated in the main text, five independent scans of the entire frequency range were conducted, and the first and fourth scans were not used for any bound-setting prior to unblinding. In practice, the first scan was used to validate the bound-setting procedures, while the fourth scan was never used. After unblinding, we decided to use the fourth scan to examine whether some peaks were transient and may have only appeared in one of the originally used datasets, something that is not expected for ALPs. Reanalyzing all excesses using only this dataset removed two-thirds of the suspicious candidates, reducing the original 3788 spikes to only 1240.

For the remaining peaks, we compared their spectral profile with the shape that is expected from an ALP. We found that most of them have a narrower spectral bandwidth than that expected from an ALP, with the majority having an ultra-narrow bandwidth, such as shown by the stars in Fig. S3. The spectral shape of an ALP is derived in Ref. [14]. To quantitatively assess their likelihood of being ALPs, we followed a simple procedure. For each spectral excess point at frequency f_0, we selected a small range of frequencies that approximately corresponded to the ALP bandwidth, as shown in red (the ALP shape is non-symmetric, so the exact condition we used was that for an ALP profile $\mathcal{A}(f)$, we took all frequencies f for which $\mathcal{A}(f) > 0.2\mathcal{A}(f_0)$). We then fitted the data to the function $\mathcal{A}(f)$ using the ALP-spin coupling g_{aNN} as a single free parameter, while neglecting the correlations of the different ALP signal frequencies. This simplification, combined with our focus on small spectral regions, means that ALP-proton and ALP-neutron interactions produce essentially identical signals (up to normalization).

To demonstrate that the fitted value is inconsistent with the prediction for an ALP, we used the fitted value of g_{aNN} to simulate a large set ($\sim 10^4$) of ALP signals. Because of their stochastic nature, we used a toy Monte-Carlo simulation to generate the ALP signals with different stochastic amplitudes (see Ref. [14] for more details). We extracted the 95% quantile of the distribution of maximal amplitudes from these simulations and compared it to the maximal amplitude in the measured data. This means that a true axion signal has a 95% chance of passing our test. Due to the poor fit of the spikes to ALPs, we found that only 5 out of the 1240 peaks satisfied this criterion. These peaks are shown in Fig. S4. 3 of these have other nearby peaks, a structure which is not expected to be present for ALPs. Only two peaks at frequencies 15.8764441 kHz and 16.11561 kHz however, remain unexplained by our additional test.

It should be noted that using a second data-set, after learning the locations and shapes of peaks, one could do a second Likelihood-based analysis to remove any peaks which are narrower than the ALP ones from their bounds, and by that improve the bound. Our pre-unblinding Likelihood-based analysis only assumed a white noise within the width of an ALP signal, but in theory, by picking the shape of the measured peaks as a background, a narrow peak should be removable from the bounds.

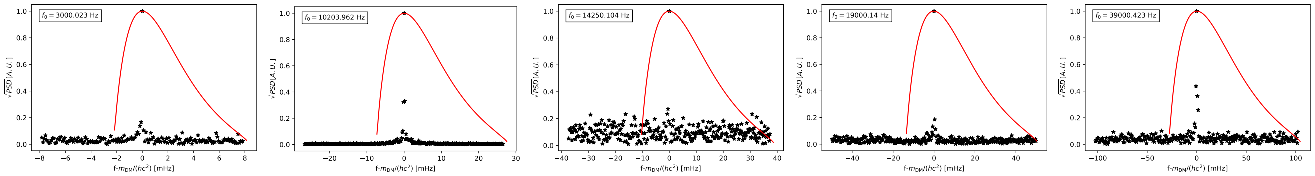

FIG. S3. **Examples of excess points that fail the original Likelihood-based analysis:** Black stars represent the square root of the Power Spectral Density (PSD), while solid red lines depict the shape of a typical ALP signal. We have normalized the peak of the ALP-signal, and the maximal $\sqrt{\text{PSD}}$ point in each figure to unity. It is evident that the signal is much wider than the observed noise. For legibility purposes, the x-axis of each plot was shifted.

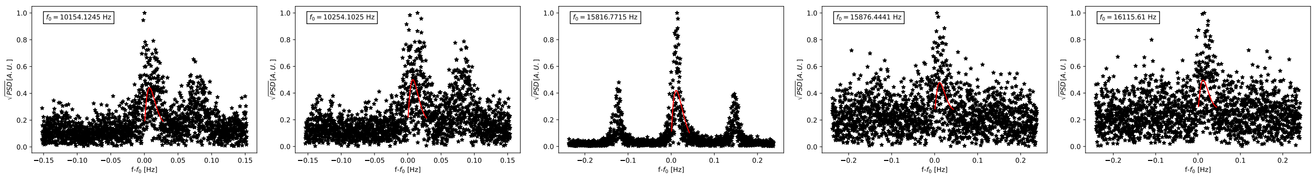

FIG. S4. **The 5 ALP candidates that failed the second test.** The square root of Power Spectral Density (PSD) is shown as black stars, and the fit for the ALP candidate is shown in red. Each noise spectrum was normalized such that its maximal $\sqrt{\text{PSD}}$ point is at unity, while the normalization of the ALP is determined from the fit to the (normalized) PSD. The x-axis of each plot was shifted for readability, with the frequency shift indicated in an inline label. Out of the 5 candidates, 3 have other peaks nearby, which suggests their entire group of nearby peaks is likely sourced by a technical noise rather than ALPs. Two candidates peaked at frequencies of 15.876441 kHz and 16.11561 kHz which are relatively isolated, remain unexplained.

1st Set	2nd Set	3rd Set	4th Set	5th Set
53.2	55.9	70.3	-54.7	70.3
9.2	52.8	68.0	-52.6	68.0
49.5	49.8	65.7	-50.4	65.7
10.7	46.8	63.5	-48.3	63.5
45.8	43.7	61.2	-46.1	61.2
12.2	40.7	58.9	-44.0	58.9
42.0	37.6	56.6	-41.8	56.6
13.7	34.6	54.3	-39.7	54.3
38.3	31.6	52.1	-37.5	52.1
15.2	28.5	49.8	-35.4	49.8
35.3	25.5	47.5	-33.2	47.5
16.6	24.0	45.2	-31.1	45.2
32.3	22.4	43.0	-28.9	43.0
18.1	20.9	40.7	-26.8	40.7
29.3	19.4	38.4	-24.6	38.4
20.4	17.9	36.1	-22.5	36.1
26.3	16.4	33.8	-20.3	33.8
23.4	14.9	31.6	-18.2	31.6
23.4	14.1	30.0	-16.8	30.0
26.3	13.3	28.5	-15.3	28.5
20.4	12.6	27.0	-13.9	27.0
29.3	11.8	25.5	-12.5	25.5
18.1	11.1	24.0	-11.0	24.0
32.3	10.3	22.4	-9.6	22.4
16.6	9.5	20.9	-8.2	20.9
35.3		19.4	-6.7	19.4
15.2		17.9	-5.3	17.9
38.3		16.4	-3.9	16.4
13.7		14.9	-2.4	14.9
42.0		14.5		14.5
12.2		14.1		14.1
45.8		13.7		13.7
10.7		13.3		13.3
49.5		13.0		13.0
9.2		12.6		12.6
53.2		12.2		12.2
		11.8		11.8
		11.4		11.4
		11.1		11.1
		10.7		10.3
		10.3		9.9
		9.9		9.5
		9.5		

TABLE S1. **The values of the external magnetic field B_z used during the search**. The values are presented in chronological order and sorted vertically in decreasing order. All values are given in units of 10^{-7} T. The first set of measurements was utilized solely to validate quality cuts and the analysis procedure, whereas the second, third, and fifth sets were used to establish the main constraints. The fourth set of measurements involved a negative sign, indicating that the direction of the magnetic field was reversed, and the field was parallel to the helium spins. This resulted in a higher EPR frequency sensed by the alkali-metal spins.

B_z	$f_{res,1}$	$f_{res,2}$	Γ_1	Γ_2	T	$1/T$	$f_{res}T$
9.5	3.0	4.1	0.8	0.65	35.3	0.028	1.3×10^8
9.9	3.1	4.4	0.74	0.7	30.2	0.033	1.1×10^8
10.3	3.3	4.6	0.79	0.6	26.5	0.038	1.0×10^8
11.1	3.8	5.1	0.75	0.59	21.2	0.047	9.4×10^7
11.4	4.0	5.3	0.85	0.69	19.3	0.052	9.0×10^7
11.8	4.2	5.5	0.78	0.72	17.6	0.057	8.6×10^7
12.2	4.5	5.7	0.77	0.71	16.3	0.061	8.3×10^7
12.6	4.7	6.0	0.83	0.66	15.1	0.066	8.1×10^7
13.0	5.0	6.1	0.82	0.77	14.1	0.071	7.8×10^7
13.3	5.2	6.4	0.85	0.8	13.2	0.076	7.6×10^7
13.7	5.4	6.5	0.73	0.83	12.5	0.08	7.4×10^7
14.1	5.6	6.8	0.73	0.76	11.8	0.085	7.3×10^7
14.5	5.9	7.0	0.81	0.78	11.1	0.09	7.2×10^7
14.9	6.1	7.2	0.8	0.8	10.6	0.094	7.1×10^7
16.4	7.0	8.1	0.85	0.9	8.8	0.113	6.7×10^7
17.9	8.0	9.0	0.79	0.83	7.6	0.132	6.4×10^7
19.4	9.1	9.9	0.78	0.81	6.6	0.151	6.3×10^7
20.9	10.0	10.9	0.78	0.79	5.9	0.17	6.1×10^7
22.4	11.0	11.8	0.8	0.78	5.3	0.189	6.1×10^7
24.0	11.9	12.7	0.9	0.82	4.8	0.208	5.9×10^7
25.5	13.0	13.7	0.87	0.85	4.4	0.226	5.9×10^7
27.0	14.0	14.6	0.9	0.84	4.1	0.245	5.8×10^7
28.5	15.1	15.5	0.87	0.87	3.8	0.264	5.8×10^7
30.0	16.1	16.5	0.89	0.88	3.5	0.283	5.8×10^7
31.6	17.1	17.5	0.89	0.88	3.3	0.302	5.7×10^7
33.8	18.7	18.9	0.9	0.89	3.0	0.33	5.7×10^7
36.1	20.2	20.4	0.93	0.91	2.8	0.359	5.6×10^7
38.4	21.7	21.8	0.93	0.93	2.6	0.387	5.6×10^7
40.7	23.2	23.4	0.94	0.93	2.4	0.415	5.6×10^7
43.0	24.7	24.8	0.95	0.94	2.3	0.444	5.6×10^7
45.2	26.2	26.2	0.95	0.96	2.1	0.472	5.6×10^7
47.5	27.6	27.7	0.98	0.97	2.0	0.5	5.5×10^7
49.8	29.1	29.1	0.99	1.01	1.9	0.527	5.5×10^7
52.1	30.6	30.6	1.04	1.03	1.8	0.555	5.5×10^7
54.3	32.1	32.2	1.06	1.06	1.7	0.584	5.5×10^7
56.6	33.6	33.7	1.05	1.08	1.6	0.613	5.5×10^7
58.9	35.1	35.1	1.06	1.05	1.6	0.639	5.5×10^7
61.2	36.6	36.6	1.08	1.07	1.5	0.668	5.5×10^7
63.5	38.1	38.1	1.1	1.09	1.4	0.695	5.5×10^7
65.7	39.6	39.7	1.11	1.1	1.4	0.725	5.5×10^7
68.0	41.2	41.2	1.11	1.11	1.3	0.753	5.5×10^7
70.3	42.8	42.8	1.14	1.14	1.3	0.78	5.5×10^7

TABLE S2. **Additional details for all measurements of the fifth set**. The table includes additional details for all measurements of the fifth set (with these details and more extractable for all measurements in Refs. [57,80,81]). The table is vertically sorted by increasing applied magnetic fields (inversely chronological). B_z is the applied external magnetic field in units of 10^{-7} T. $f_{res,1}$ ($f_{res,2}$) is the resonance frequency measured at the pre-measurement (post-measurement) calibration in units of kHz. Γ_1 (Γ_2) is the fitted response width, measured at the pre-measurement (post-measurement) calibration in units of kHz. T is the total time duration of the measurement in units of 10^3 seconds. $1/T$ is the inverse of the total time duration of the measurement in units of mHz (and is also the frequency resolution of the computed Fourier transform). $Tf_{res} \equiv T(f_{res,1} + f_{res,2})/2$ is the number of oscillations that would measured in the time duration for a typical frequency to which the measurement was sensitive. The sampling rate at all measurements was 100 kHz. Note that both Γs are the fitted widths, rather than the width of the Lorentzian response, and hence at low frequencies ($\lesssim 5$ kHz), they are affected by the Lorentzian centered around $-f_{res}$ as well as the non-trivial detector response, and the decay of the helium spins during the calibration, and are therefore larger than what would be the fundamental Γ of the alkali. See section S1 for further details.
